# Serum Metabonomic Analysis of Protective Effects of *Curcuma aromatica* Oil on Renal Fibrosis Rats

**DOI:** 10.1371/journal.pone.0108678

**Published:** 2014-09-29

**Authors:** Liangcai Zhao, Haiyan Zhang, Yunjun Yang, Yongquan Zheng, Minjian Dong, Yaqiang Wang, Guanghui Bai, Xinjian Ye, Zhihan Yan, Hongchang Gao

**Affiliations:** 1 Institute of Metabonomics & Medical NMR, School of Pharmaceutical Sciences, Wenzhou Medical University, Wenzhou, China; 2 Radiology Department of the Second Affiliated Hospital, Wenzhou Medical University, Wenzhou, China; 3 Department of Radiology, the first Affiliated Hospital, Wenzhou Medical University, Wenzhou, China; Jawaharlal Nehru University, India

## Abstract

**Background:**

*Curcuma aromatica* oil is a traditional herbal medicine demonstrating protective and anti-fibrosis activities in renal fibrosis patients. However, study of its mechanism of action is challenged by its multiple components and multiple targets that its active agent acts on.

**Methodology/Principal Findings:**

Nuclear magnetic resonance (NMR)-based metabonomics combined with clinical chemistry and histopathology examination were performed to evaluate intervening effects of *Curcuma aromatica* oil on renal interstitial fibrosis rats induced by unilateral ureteral obstruction. The metabolite levels were compared based on integral values of serum ^1^H NMR spectra from rats on 3, 7, 14, and 28 days after the medicine administration. Time trajectory analysis demonstrated that metabolic profiles of the agent-treated rats were restored to control levels after 7 days of dosage. The results confirmed that the agent would be an effective anti-fibrosis medicine in a time-dependent manner, especially in early renal fibrosis stage. Targeted metabolite analysis showed that the medicine could lower levels of lipid, acetoacetate, glucose, phosphorylcholine/choline, trimethylamine oxide and raise levels of pyruvate, glycine in the serum of the rats. Serum clinical chemistry and kidney histopathology examination dovetailed well with the metabonomics data.

**Conclusions/Significances:**

The results substantiated that *Curcuma aromatica* oil administration can ameliorate renal fibrosis symptoms by inhibiting some metabolic pathways, including lipids metabolism, glycolysis and methylamine metabolism, which are dominating targets of the agent working *in vivo*. This study further strengthens the novel analytical approach for evaluating the effect of traditional herbal medicine and elucidating its molecular mechanism.

## Introduction

Renal interstitial fibrosis (RIF) is the common end of progressive chronic renal disease. It is a complex pathologic process involving inflammation, lipid peroxidation, oxidative stress, hypoxia, also is characterized by cellular infiltration, fibroblast differentiation, increased extracellular matrix protein deposition, and tubule atrophy [1]. Once renal disease progresses to end-stage renal disease, patient survival will only depend on dialysis and kidney transplantation, which bring enormous physical and psychological pain, not to mention high economic burden. Thus, blocking or reversal of the progressive course of renal fibrosis is a key to the prevention of the disease advancing to end-stage. Although numerous efforts have been exerted to develop therapeutic strategies, such as angiotensin converting enzyme inhibitors and angiotensin II receptor blockers, fibrotic renal disease has remained a major unresolved problem in the clinical therapy of renal disease.

Traditional herbal medicines have been gaining more attention worldwide due to their intrinsic unique advantages, such as a holistic approach to examining the function and dysfunction of living organisms, different from the so-called “Western” medicine [2]. Our previous study proved that *Curcuma aromatica* oil (CAO), the volatile oil substances of the medicine, significantly attenuated total collagen of renal fibrosis rats after a 14-day treatment [3]. The agent was also found to ameliorate symptoms of diabetic nephropathy in rats [4]. Utilizing molecular biology technology, crystallization ingredients of *Curcuma aromatica* were certified to demonstrate protective activities against renal fibrosis through the up-regulation of heme oxygenase [5] and through the Nrf2-Keap1 signal transduction pathway [6]. As a traditional herbal medicine, *Curcuma aromatica* is gaining more attention worldwide due to its long history of use in clinical practice. However, as *Curcuma aromatica* usually operates in the body through multiple components, multiple pathways, and multiple targets, not to mention the complex pathological process of renal fibrosis itself, the study of its anti-fibrosis mechanism is challenging.

Metabonomics technology, a non-targeted analysis of tissues or biofluids for organic low-molecular metabolite, provides entire metabolic profiles of organisms undergoing medical intervention and offers novel insight on the understanding of pharmacological mechanism [7]. Metabonomic analysis has been applied to evaluate therapeutic effects of many traditional herbal medicines, including *Rhizoma drynariae* extracts [8], berberine [9], *Shuanglong* formula [10], and *Liuwei Dihuang* pills [11]. Using NMR-based metabonomics, we previously evaluated the therapeutic effect of *Zhibai Dihuang* pill in the treatment of diabetic nephropathy rats [12]. These previous studies illustrated that metabonomics analysis is an effective approach for evaluating the effects of and for elucidating the mechanism behind traditional herbal medicine [13], [14].

In this work, we used unilateral ureteral obstruction (UUO)-induced rats as a RIF model developing renal injury similar to clinical human renal fibrosis [15]. NMR-based metabonomics was applied to analyze the metabolic profile changes in the serum of the control, UUO, and CAO-treated RIF rats at different time points. We sought to determine (*i*) whether the metabolic profiles of RIF rats could be ameliorated by CAO treatment, (*ii*) which of the metabolic pathways disturbed in RIF rats could be intervened in by CAO treatment, and (*iii*) how the metabolic mechanism of CAO acts on holistically *in vivo*. The results of our study can facilitate the understanding of the regulation of traditional Chinese medicine, as well as its molecular mechanisms.

## Materials and Methods

### Subjects and materials

CAO was produced by the Tian Rui Medicine Co., Ltd. (Zhejiang, China). Adult male Sprague-Dawley rats (8 weeks age, 220±15 g), obtained from Shanghai SLAC Laboratory Animal Co. Ltd. (Shanghai, China), were kept in specific pathogen-free (SPF) colony of Laboratory Animal Center of Wenzhou Medical University, with regulated temperature and humidity and a 12/12 h light-dark cycle with lights on at 08:00 am. The animal production licenses are No. SCXK 2007-0005. During the whole experimental process, rats were fed with standard rat chow and tap water.

### Ethics statement

The protocol for the animal experiment was approved by the Institutional Animal Committee of Wenzhou Medical University. All animals received care in accordance to the ‘Guide for the Care and Use of Laboratory Animals’. Procedures using rats were approved by the Institutional Animal Care and Use Committee of Wenzhou Medical University (document number: wydw2012-0083).

### Experimental design and sample collection

After acclimation of one week prior to conducting experiments, the rats were randomly divided into three groups: sham-operation control group (SO group), UUO model group (UUO or RIF group) and CAO-treated RIF group (CAO group), *n* = 8 for each group and 6–8 serum samples were obtained at each time point (3 d, 7 d, 14 d, and 28 d after UUO model was founded). In addition, to conclude that CAO treatment effect on serum profile is specific to renal fibrosis and to assist understanding the effect of CAO on the healthy rats, we also performed one sham-operated CAO-treated control group (CAO-SO group). The UUO and CAO rats were conducted to UUO model according to the operating procedure described previously [16]. Rats were anesthetized by i.p. injection of 10% chloral hydrate (3 mL/Kg), then, the left ureter was isolated and completely ligated with 4-0 silk sutures. The SO and CAO-SO rats underwent an identical surgical procedure without ureteral ligation. Rats were allowed to recover from anesthesia and were housed in standard rodent cages with ad libitum access to water and food until sacrificed. Solid food was withdrawn 12 h before and after surgery.

Three different doses of CAO treatment on RIF rats (100 mg/kg, 200 mg/kg, 300 mg/kg body weight, *i.p.*) were utilized in order to determine its optimal usage. The biochemical analysis and histopathology, as shown in Table S1 and Figure S1, suggest obvious amelioration of biochemical parameters and histopathology damages in RIF rats at the two high doses. Interestingly, negligible difference was found between higher doses, i.e. 200 mg/kg versus 300 mg/kg. And, refer to our previous study [3], we chose the dosage of 200 mg/kg in the subsequent studies, in which the drug-treated rats were administrated with CAO (200 mg/kg, *i.p.*) every 3 days for the following 28 days. The UUO and SO rats were administrated with the same volume of vehicle.

1.5 mL blood samples were drawn from the suborbital vein at days 3, 7, 14 and 28 post-operation under 10% chloral hydrate induced anesthesia. The sera were separated after centrifugation at 3 000 g for 10 min at 4°C and then stored at −80°C until NMR and clinical chemical analysis. Before sacrifice, the left kidneys were removed from the anesthetic rats and immersed in 10% (w/v) neutral buffered formalin solution for histological analysis.

### Clinical chemistry and histopathology analysis

The clinical chemistry analysis of serum was carried out for the measurement of biochemical parameters including serum creatinine (SCr), blood urea nitrogen (BUN), triglycerides (TG), low density lipoproteins (LDL), and high density lipoproteins (HDL) by using Automatic Analyzer (BECKMAN-COULTER LX-20, USA). Histopathological changes in kidney tissues were assessed in at least 25 randomly selected tissue sections from each group under study. Sections were stained with Mayer's haematoxylin and eosin (HE) staining for light microscopic observation.

### Preparation of samples and acquisition of ^1^H NMR spectra

Prior to NMR analysis, serum samples were thawed and 200 µL of each was diluted by 400 µL of phosphate buffer (0.2 mM Na_2_HPO_4_/NaH_2_PO_4_, pH 7.4) to minimize variations in pH and 60 µL D_2_O for a field frequency lock. The mixed serum samples were centrifuged at 12 000 g for 10 min at 4°C to separate any precipitate. Aliquots of 500 µL of the supernatants were transferred to 5 mm NMR tubes. The detailed protocol refers to our previous work [17].

All NMR spectra were recorded at 298 K on a Bruker AVANCE III 600 NMR spectrometer operating at 600.13 MHz ^1^H frequency and equipped with a triple resonance probe. The CPMG (Carr-Purcell-Meiboom-Gill) pulse sequence was used to attenuate the broad NMR signals from slowly tumbling molecules such as proteins and retain those from low-molecular weight compounds and some lipid components with a fixed relaxation delay 2nτ of 120 ms. Typically, 128 scans were collected into 64 K data points over a spectral width of 12 000 Hz with a relaxation delay of 4 s and an acquisition time of 2.65 s. An exponential line-broadening of 0.3 Hz was applied to the FID prior to Fourier transformation. All spectra were carefully phased and baseline corrected and referenced to the methyl peak of lactate (CH_3_, δ1.33) [18].

### Multivariate pattern recognition

Following phase and baseline correction, each spectrum was segmented into chemical shift regions of equal width of 0.0015 ppm (buckets) corresponding to the region of δ 10.0 to 0.4 using the Bruker Topspin 2.1 software package. The region of δ 5.2 to 4.4 (containing the residual peak from the suppressed water resonance) was set to the zero integral in the analysis. The remaining spectral segments for each NMR spectrum were normalized to the total sum of the spectral intensity to partially compensate for differences in concentration of the many metabolites in the samples, and the normalized integral values were entered into SIMCA-P+12.0 software (Umetrics, Umeå, Sweden) for principal component analysis (PCA) and partial least squares-discriminant analysis (PLS-DA).

PCA is unsupervised multivariable statistical method, in which data are visualized by plotting the scores of the first two principal components (PC1 and PC2) to provide the most efficient 2D representation of the information, where the position of each point along a given axis in the scores plot is influenced by variables in the same axis in the loading plot. As a supervised multivariable statistics, PLS-DA revealed differences in the different groups, which is necessary to eliminate outliers and enhance the quality of the PCA model. Leave-one-out cross validation and permutation tests (200 cycles) were conducted to measure the robustness of the model obtained because of the small number of samples. The quality of the PCA and PLS-DA model is described by R^2^ and Q^2^ values. R^2^ is defined as the proportion of variance in the data explained by the model, indicating goodness of fit. Q^2^ is defined as the proportion of variance in the data predicted by the model, indicating predictability [19]. Loading plots were used to identify the metabolites responsible for the separation of groups. Significant differences among metabolites in PLS-DA were assessed by the absolute value of correlation coefficient, |r|, which was calculated in a Java environment.

### Statistical analysis

To detect significant differences between metabolic changes, the normalized integral values of 0.0015 ppm were input into SPSS software (version 13.0, SPSS Inc, USA) for statistical analysis. Data were first analyzed using one-way analysis of ANOVA followed by the Dunnett's test (two-tailed). *P* value of less than 0.05 was considered statistically significant.

## Results

### Clinical chemical analysis and histopathology

Results of the biochemical analysis are depicted in **Table 1**. Compared with SO rats, distinct increase in serum LDL and HDL of UUO rats was observed at all investigated time points. For CAO rats, serum LDL concentration was significantly reduced by more than 50% of the level of UUO rats, whereas HDL concentration remained stable. Especially, after drug treatment for 7 days, almost all lipid indexes returned nearly to control levels, including TG. Indicators of renal (glomerular) function, SCr, and BUN levels were ameliorated markedly in CAO rats compared with UUO rats. While, these serum biochemical parameters were almost kept unchanged in CAO-SO rats. This suggested that renal function could be recovered by CAO agent dosage and the CAO treatment effect is specific to renal fibrosis. Interestingly, the SCr levels in CAO treated rats decreased significantly compared with the SO rats, which suggests that CAO modifying the creatinine metabolism might contribute a part of the mechanisms associated with CAO ameliorating renal fibrosis symptoms.

**Table 1:** Serum clinical chemistry parameters.

	3 d			7 d			
	SO	UUO	CAO-UUO	SO	UUO	CAO-UUO	CAO-SO
LDL (mM/L)	1.68±0.58	2.38±0.87*	1.61±0.46#	1.25±0.26	2.62±0.46**	1.89±0.33#	1.27±0.62
HDL (mM/L)	0.40±0.17	0.93±0.56*	0.92±0.37	0.30±0.12	1.52±0.58**	1.12±0.48	0.27±0.08
TG (mM/L)	0.69±0.33	0.68±0.24	0.85±0.47	0.51±0.12	0.77±0.37	0.71±0.35	0.70±0.26
BUN (mM/L)	8.47±2.36	8.87±2.10	8.45±1.45	10.56±1.98	8.55±1.80	11.36±3.18#	9.85±3.45
SCr (umol/L)	79.23±9.37	102.90±27.67*	47.59±14.45* #	70.73±26.18	99.80±17.13**	30.78±7.35* ##	28.10±6.15*

	14 d			28 d		
	SO	UUO	CAO-UUO	SO	UUO	CAO-UUO
LDL (mM/L)	1.51±0.57	2.16±0.45*	1.54±0.28#	1.35±0.91	2.09±0.29*	1.93±0.25
HDL (mM/L)	0.53±0.11	1.89±0.40**	1.05±0.67#	0.33±0.13	1.95±1.21*	1.39±0.47
TG (mM/L)	0.69±0.42	0.91±0.36*	0.57±0.22##	0.61±0.31	0.61±0.37	0.56±0.23
BUN (mM/L)	7.14±2.68	9.78±2.72*	7.11±2.75#	8.97±1.08	11.60±0.88**	6.13±2.28##
SCr (umol/L)	73.47±24.34	79.93±21.67	46.40±10.07* #	96.15±9.39	104.23±25.44	42.22±7.38* ##

Data are expressed as mean ± SD (n = 6–8 for each group). LDL, low density lipoproteins; HDL, high density lipoproteins; TG, triglycerides; BUN, blood urea nitrogen; SCr, serum creatinine.

*P<0.05,

**p<0.01, compared with SO rats.

# P<0.05,

## p<0.01, compared with UUO rats.

**Figure 1:**
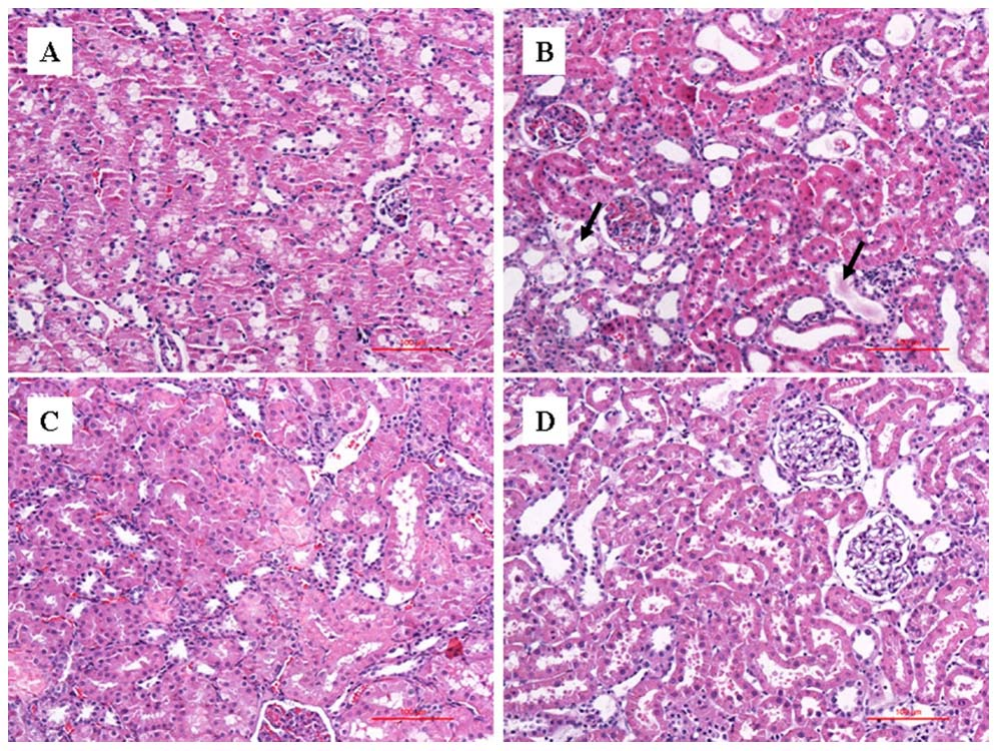
Representative HE-stained sections (200 fold) of kidneys from SO (A), UUO (B), CAO rats on 7 d (C) and 28 d (D), respectively. The SO rats exhibit no abnormal histopathological changes, whereas UUO rats display loss of brush border, tubular swelling atrophy and cystic dilation. Meanwhile, CAO rats display the different therapeutic effects of the agent.

**Figure 1** shows the representative images of the histological examination of HE-stained sections of kidneys from three groups of rats. SO rats does not exhibit abnormal histopathological changes (**Figure 1A**), whereas UUO rats display, to some extent, loss of brush border, interstitial inflammation, tubular swelling atrophy, and cystic dilation at both time points (**Figure 1B**). These findings suggested tubular injuries and fibrotic process in the kidney tissues from the rats during the whole experimental period. Meanwhile, CAO treatment significantly attenuated tubular damages in RIF rats on day 7, with the subjects showing no signs of tubular denaturation or necrosis, similar to the normal organizational structure of SO rats (**Figure 1C**). Interestingly, an apparent increase in tubular lesions and interstitial myofibroblasts were observed in CAO rats on day 28, similar to RIF rats (**Figure 1D**). Significant differences of the recovery trends among CAO-administrated rats illustrated the time-dependent therapeutic effects of CAO on RIF rats.

### Time-dependent effects of CAO on the metabolic pattern of RIF rats

**Figure 2:**
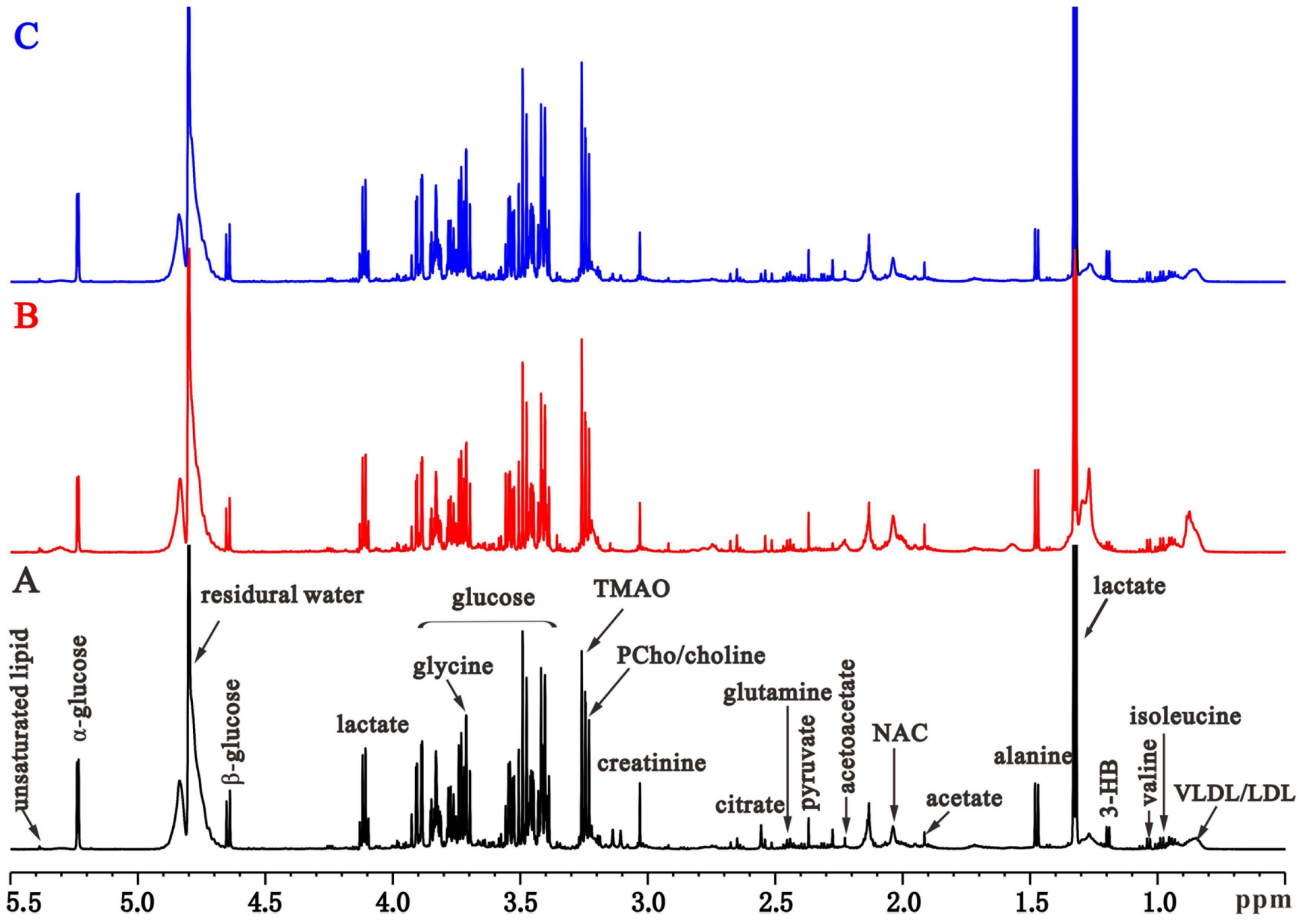
Representative ^1^H CPMG spectra of serum obtained from the SO (A), UUO (B) and CAO (C) rats on day 7, respectively. NAC, N-acetylglycoproteins; TMAO, trimethylamine-N-oxide; 3-HB, 3-hydrobutyrate.

Representative ^1^H NMR spectra of the serum samples obtained from SO, UUO, and CAO groups are shown in **Figure 2**. Resonance assignments were performed based on two-dimensional ^1^H-^1^H COSY and TOCSY spectra (data not shown) and our previous studies [20], [21]. Firstly, to access changes of the metabolites concentration over time, the integral levels of serum samples from all the groups of the rats on days 3, 7, 14, and 28 were compared in **Table 2**. On both time points of day 7 and day 14, UUO rats showing increased levels of low-density lipoproteins/very low-density lipoproteins (LDL/VLDL), valine, leucine, isoleucine, alanine, acetate, trimethylamine-N-oxide (TMAO), creatine, and phosphatidylcholine/choline (PCho/choline), as well as decreased levels of 3-hydrobutyrate (3-HB), pyruvate, and glycine, comparing to the SO rats. Disturbances in endogenous metabolite levels were different between the earlier and later stages of RIF, and the trends were in good agreement with our previous results [22]. Interestingly, after CAO administration, disturbances in endogenous metabolite levels of UUO rats were almost recovered towards the control levels, especially on day 7 and day 14 timepoints. The results were supported by clinical chemistry and histopathology data, which substantiated that a time-dependent therapeutic effect of the agent in the body.

**Table 2:** Summary of change trends of metabolites significantly increased/decreased compared to SO or UUO groups in relative integral levels.

δ^1^H (ppm)	Metabolite	3 d		7 d		14 d		28 d	
		U vs. S	C vs. U	U vs. S	C vs. U	U vs. S	C vs. U	U vs. S	C vs. U
0.86,1.26	LDL/VLDL	↑*	—	↑*	↓*	↑*	↓*	↑*	↓*
0.94	Isoleucine	—	—	↑*	—	↑*	—	↑*	—
1.04	Valine	—	—	↑*	—	↑*	—	↑*	—
1.19	3-HB	↓*	—	↓*	↑*	↓*	↑*	↑*	↓*
1.32,4.10	Lacate	↓*	—	↓*	↑*	↑*	↓*	↑*	↓*
2.14	Acetoacetate	—	—	↑*	↓*	↑*	↓*	↑*	↓*
2.34	Pyruvate	—	↓*	↓*	↑*	↓*	↑*	↑*	↓*
2.52	Citrate	↓*	—	—	—	—	—	↑*	—
3.22	Pcho/choline	—	—	↑*	↓*	↑*	↓*	↓*	↓*
3.26	TMAO	↑*	↓*	↑*	↓*	↑*	↓*	↓*	—
3.54	Glycine	—	—	↓*	↑*	↓*	↑*	↓*	—
3.70–3.90	Glucose	—	↓*	↑*	↓*	↑*	↓*	↑*	↓*
5.30	Unsaturated lipid	↑*	—	↑*	↓*	↑*	↓*	↑*	↓*

S, sham-operation control group; U, unilateral ureteral obstruction group; C, Curcuma aromatica oil-treated group. 3-HB, 3-hydrobutyrate; TMAO, trimethylamine-N-oxide. Marks indicate the direction of the change, i.e. ↓ for decrease, ↑ for increase, – for no change.

*P<0.05 and

**P<0.01.

**Figure 3:**
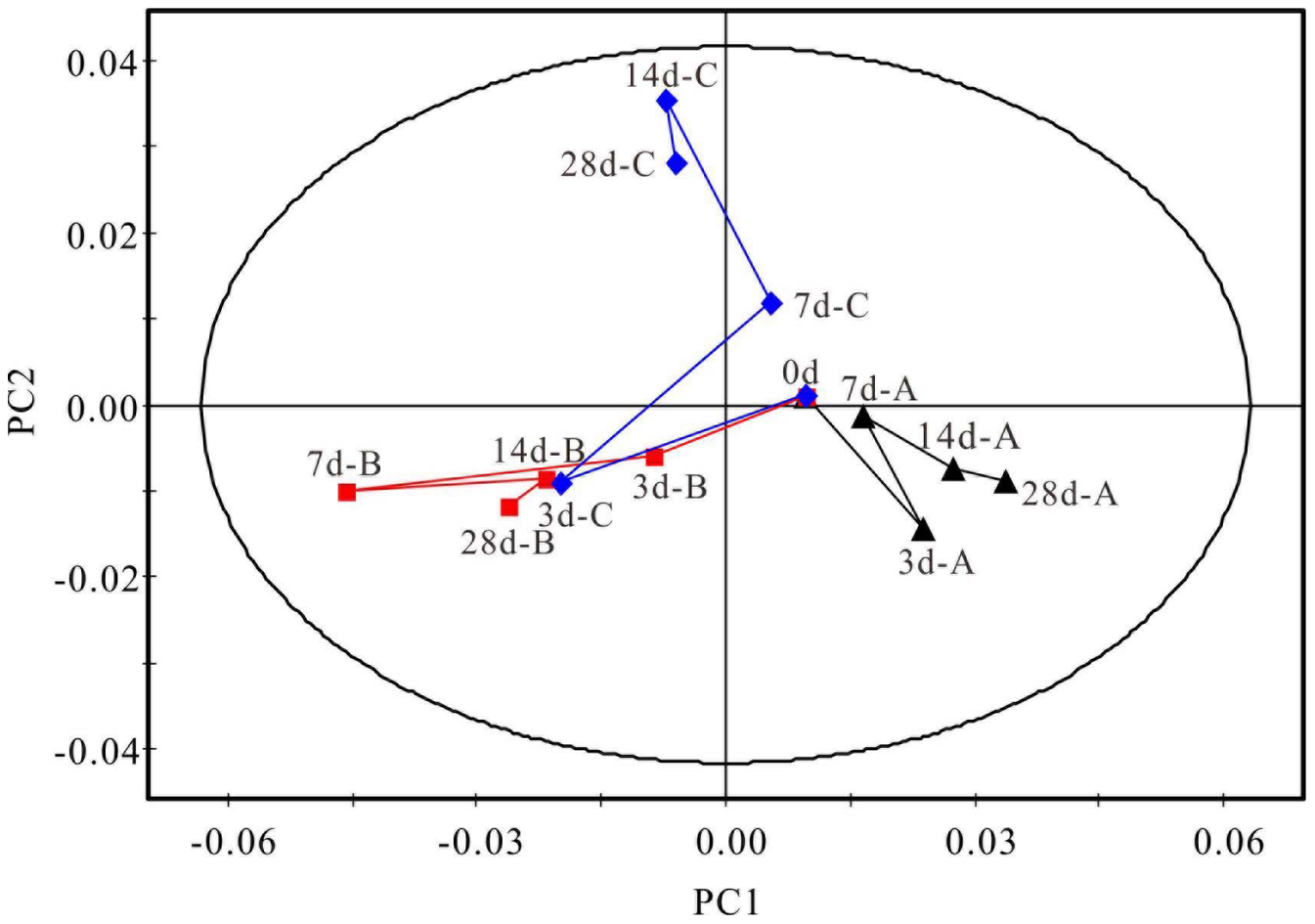
Time trajectory plot of mean data of serum ^1^H CPMG spectra from SO (▴A), UUO (▪B) and CAO (⧫C) rats, respectively. The plot suggests time-dependent metabolic characteristics of different group rats.

Then, to further evaluate the time-dependent interventional effect of CAO on renal fibrosis, average integral values of ^1^H NMR spectra of all the serum samples were subjected to PLS-DA (**Figure 3**, R^2^X = 0.844, Q^2^ = 0.738), by which a pairwise day-to-day comparison can provide more details on the pathological process of the RIF. In the plot, a clear separation of trajectory diagrams of the UUO and SO groups was displayed, and the CAO group was almost located separated from UUO and SO groups. On day 7, the metabolic pattern of CAO rats ultimately approached to the region of SO rats, which suggested that the CAO treatment effect is significant to renal fibrosis on that timepoint. Interestingly, on days 14 and 28, while still located between the UUO and SO rats, the spot of CAO rats shifted farther from SO rats than that on day 7. All the data illustrated the renal fibrosis symptom of the RIF rats was prevented and alleviated by CAO treatment, and that maximum therapeutic efficacy of the agent was achieved after the drug administration for 7 days.

### Metabonomic analysis of the CAO effect to RIF rats on day 7

**Figure 4:**
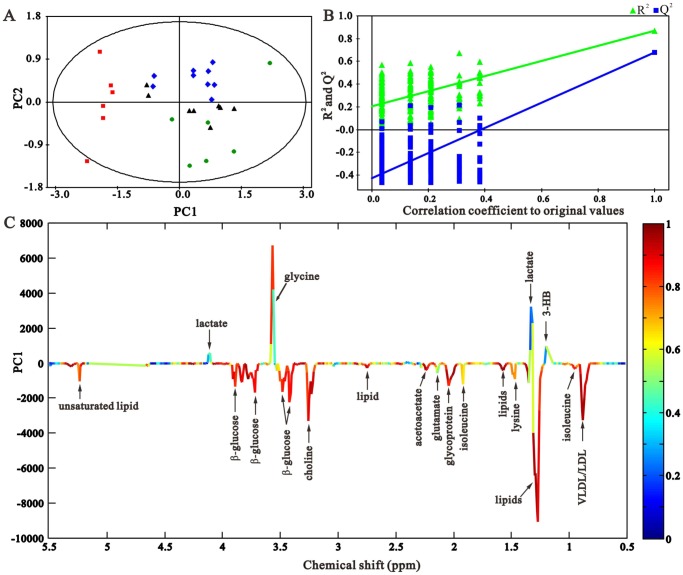
PCA scores plot (A), validation plot (B) and coefficient-coded loading plot (C) based on the ^1^H CPMG spectra of control (▴), UUO (▪), CAO-UUO (⧫) and CAO-SO (•) rats on day 7. The coefficient-coded loading plot corresponding to PCA revealing the metabolites with large intensities responsible for the discrimination of the corresponding score plots.

To further explore the differences in the metabolic profiles of the treated groups, we subjected the samples of day 7 to PCA (**Figure 4**). We found that serum samples of CAO treated-SO rats were mixed in the cluster of SO rats, demonstrating that CAO treatment did not obviously alter the serum profile of SO rats. And CAO treated-UUO rats were clustered in the SO group, and separated with UUO group obviously (**Figure 4A**, R^2^X = 0.93, Q^2^ = 0.835), suggesting that dosage of the agent induced the metabolic profiles of the RIF rats to recover toward that of the control. This is in good agreement with the data of clinical biochemical analysis displayed above. **Figure 4B** shows the validation plot of permutation tests showed that the PCA model is robust and credible. **Figure 4C** illustrates the corresponding loading plot with color-coded correlation coefficients (|r|) of metabolites between different groups and shows the variables responsible for the separation of them. The weight of a variable in the discrimination is given by the square of its correlation coefficient, which is color-coded from zero in blue to high values in red. The negative regions in the loading plot corresponded to metabolites that increased in quantity in the serum of the rats, whereas positive regions corresponded to metabolites that decreased in quantity in the serum of rats.

**Figure 5:**
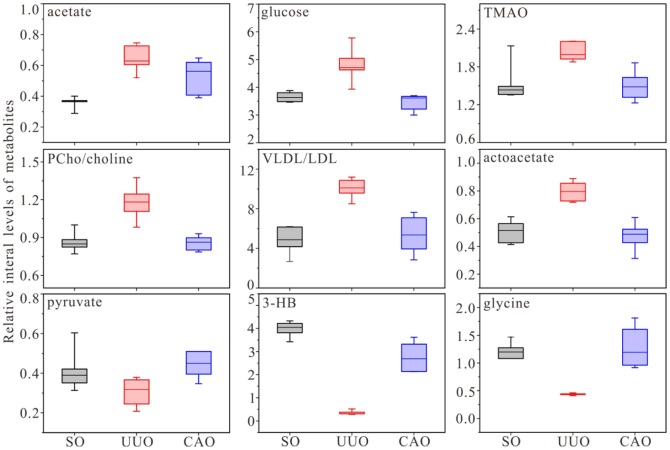
Box plots of relative integral levels of metabolites in serum samples of different groups of rats on day 7. The plots show that most of metabolic levels altered in UUO rats, are recovered to control level after CAO treatment.

**Figure 6:**
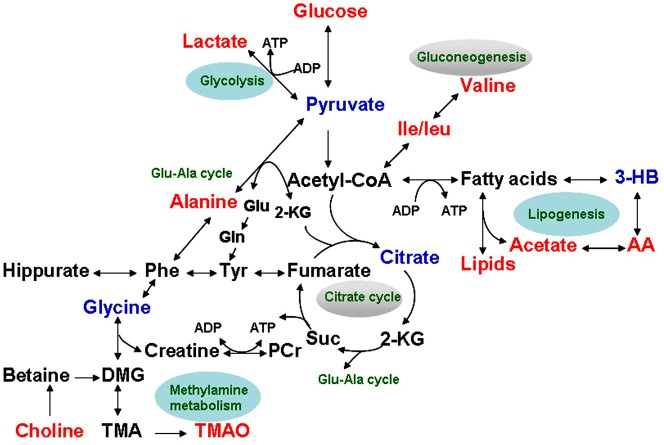
Disturbed metabolic pathways in serum of RIF rats treated with CAO. In UUO rats metabolites with increased and decreased levels compared to control rats are indicated in red and blue.

To further verify the observed restoring effects of CAO treatment on RIF rats, box plots for typical metabolites concentration of serum samples on day 7 are displayed in **Figure 5**. Compared with that in UUO rats, most metabolites revealed manifest tendencies of recovery toward SO level in CAO rats, except for citrate, valine, leucine, and isoleucine (data not shown). Then the variations of metabolites allowed us to explore some important information about the mechanisms involved in molecular mechanism of the agent acting on renal fibrosis. **Figure 6** illustrates the altered metabolic pathways during the development of the disease, and drug-regulated targets based on the KEGG database (http://www.genome.jp/kegg/pathway.html).

## Discussion

As a traditional herbal medicine, *Curcuma aromatica* has been widely used to treat arthritic pain, gastrointestinal, fibrosis, cancer in Asian countries such as India, Thailand, and China for a long time [23]. Modern medical research have demonstrated that *Curcuma aromatica* contains anti-inflammation, anti-oxidation, anti-viral, and anti-carcinogenic ingredients [23], [24]. However, due to the multiple substances that comprise *Curcuma aromatica*, the detailed molecular mechanism of the active component in the body remains unclear. Metabonomics is an effective approach for evaluating the therapeutic effects of traditional herbal medicine. By identifying global metabolite levels and metabolic pathways after agent intervention, the approach opens up the possibility of studying the effect of multiple mixtures in complex biological systems, thereby bridging systems biology and traditional herbal medicine [2]. Thus, in this study, we applied quantitative metabonomics analysis to evaluate the therapeutic effect of CAO on RIF rats and to elucidate its underlying molecular mechanism.

In this study, the histopathological and metabolic analyses described above showed that treatment of CAO can prevent renal tissues from fibrosis injury in the early stage of RIF. Furhtermore, a pairwise day-to-day comparison by PLS-DA confirmed that CAO has the most manifest therapeutic effect on day 7 timepoint when the renal damage is mild. The metabolites altered and related pathways that appear to be regulated by CAO may represent potential metabolic biomarkers for disease diagnosis and provide clues to elucidate the molecular mechanism of the agent acting on renal fibrosis (**Figure 6**).

### Lipids metabolism

Renal interstitial fibrosis is a multistep process involving inflammation, lipid nephrotoxicity, hypoxia, and oxidative stress. In this study, both biochemical and NMR analyses showed elevated concentration of VLDL/LDL, acetate, and triglycerides, as well as reduced level of 3-HB and acetoacetate (ketone body metabolism), in UUO rats. Excessive lipid accumulation in the blood of UUO rats stimulates the conversion of fatty acids into acetyl-CoA through the process of β-oxidation. As an important intermediate product of glucose and lipid metabolism, acetyl-CoA enters the TCA cycle or condenses into ketone bodies through which it is oxidized to supply the body with energy. Increased levels of VLDL/LDL and TG in the blood may be related to the reduction pathways of ketone bodies and TCA cycle metabolism in disease state. It was confirmed that hyperlipidemia had a profibrotic function in the pathogenesis of RIF [25]. In addition, as markers of medullar injury in the pathogenesis of kidney disease, increased PCho/choline and TMAO concentrations suggest enhanced N-oxidation of fatty acids in UUO rats [26]. The results of the study demonstrated that CAO dosage decreased the levels of TMAO, PCho/choline, and lipid metabolites in the serum, indicated that CAO may provide additional beneficial effects on renal injuries by lowering lipids levels in RIF rats.

### Glucose metabolism

In earlier stages of RIF, the increased concentrations of glucose and lactate, as well as the decreased levels of pyruvate and citrate in the serum, are indicative of enhanced glycolysis and inhibited glucose oxidation process in model rats. Pyruvate, an important intermediate product of glycolysis and TCA cycle, is frequently associated with glycometabolism. It can be used to produce acetyl-CoA by the pyruvate dehydrogenase complex, after which it enters the TCA cycle, or it could be converted into alanine via alanine aminotransferase, into lactate via lactate dehydrogenase. It was reported that in the case of RIF, a large amount of acetyl-CoA was generated, which would exert a negative feedback on the activity of pyruvate dehydrogenase complex, thus inhibiting the pathway of TCA cycle and allowing the domination of two other outputs (lactate and glucogenic amino acids) [27]. Also the inhibited TCA cycle may be related to an impaired mitochondrial function in the proximal tubule of the RIF rats [28], [29].

The enhanced pathway of the glycogenic amino acids (e.g., leucine, isoleucine and valine) metabolism possibly suggests tubular damage-induced protein degradation or disturbed protein synthesis [30]. In this study, decreased levels of pyruvate and citrate and elevated levels of lactate and certain glycogenic amino acids in earlier UUO stage indicate that glucose aerobic oxidation was inhibited and that the energy consumption pattern may have shifted to glycolysis and gluconeogenesis in response to RIF. Interestingly, after CAO treatment, the levels of glucose, lactate, and pyruvate recovered to control levels, whereas the concentration of citrate and glycogenic amino acids exhibited no change. These results suggest that CAO operates *in vivo*, probably by affecting the glycolysis pathway as opposed to the glucose oxidation and gluconeogenesis pathways.

### Methylamine metabolism

Functioning as a conjugation acid of acetyl-CoA synthetase, glycine can protect renal cells from ATP depletion-induced injury [31], [32] and keep proximal tubules from suffering from hypoxia, inflammation, and interstitial fibrosis [33]. The reduced level of glycine, and elevation of SCr and BUN in UUO rats as revealed by biochemical examination indicate the dysfunction of tubular reabsorption and perturbation in the methylamine metabolism pathway. Several other pivotal product concentrations, such as choline, creatine and TMAO discussed above, were enhanced in the serum of UUO rats. The increased levels of metabolites in the methylamine pathway in the disease state were restored toward control levels after CAO treatment, which supports the protective effects of the agent on renal tubular function by regulating the methylamine metabolism pathway.

## Conclusions

In this work, NMR-based serum metabonomics analysis was applied to evaluate the effects of CAO dosage on RIF rats and to elucidate the underlying protective molecular mechanism. CAO treatment was found to significantly ameliorate renal fibrosis symptoms in time-dependent manner, especially in earlier RIF stage, by intervening in some dominating metabolic pathways, including glycolysis, lipid metabolism and methylamine metabolism. However, the other metabolic pathways altered in UUO rats, such as the gluconeogenesis and TCA cycle, were not ameliorated correspondingly in CAO-treated rats. Our results identified the main targets of the *Curcuma aromatica* agent that worked in the body, which need to be further studied with other complementary approaches including molecular biology and enzyme activity determination. This work also contributes to the evaluation of metabonomic technology on the efficacy of traditional herbal medicine and its underlying molecular mechanism.

## Supporting Information

Figure S1 **Representative HE-stained sections (200 fold) of kidneys from UUO (A), CAO doses of 100 mg/kg (B), 200 mg/kg (C) and 300 mg/kg (D) for 7 days, respectively.** The UUO rats display clearly loss of brush border, tubular swelling atrophy and cystic dilation, while such histopathology damages were somewhat ameliorated in the CAO rats.
(EPS)

Table S1 **Serum clinical chemistry parameters of different dosages of CAO on RIF rats for 7 days.**
(DOCX)
