# Supplementary material for: Serum Metabonomic Analysis of Protective Effects of Curcuma aromatica Oil on Renal Fibrosis Rats
Source: PLoS One. 2014 Sep 29;9(9):e108678. doi: 10.1371/journal.pone.0108678 (PMC4181651; doi:10.1371/journal.pone.0108678)
Supplement: Table S1 — Serum clinical chemistry parameters of different dosages of CAO on RIF rats for 7 days. (DOCX) [file pone.0108678.s002.docx]

**Table S1.** Serum clinical chemistry parameters of different dosages of CAO on RIF rats for 7 days.

|  |  |  |  | CAO groups | |  |
| --- | --- | --- | --- | --- | --- | --- |
| Indicators | SO | UUO | 100 mg/kg | 200 mg/kg | 300 mg/kg | |
| LDL (mM/L) | 1.32±0.48 | 2.76±0.79^*^ | 2.74±0.77^*^ | 1.75±0.64^*##^ | 1.65±0.52^*##^ | |
| HDL (mM/L) | 0.60±0.24 | 1.63±0.41^*^ | 1.31±0.44^*^ | 0.93±0.46^*##^ | 0.88±0.50^##^ | |
| TG (mM/L) | 0.82±0.38 | 0.99±0.25 | 0.88±0.27 | 0.81±0.27 | 0.69±0.37 | |
| BUN (mM/L) | 8.62±1.96 | 8.78±2.51 | 10.88±1.87^#^ | 11.91±2.22^#^ | 12.61±3.37^##^ | |
| SCr (umol/L) | 81.43±12.55 | 116.22±27.00^*^ | 54.13±18.43**^##^ | 39.69±10.23**^##^ | 30.24±10.04**^##^ | |

Data are expressed as mean ± SD (n = 4 for each group). CAO, *Curcuma aromatica* oil; RIF, Renal interstitial fibrosis; LDL, low density lipoproteins; HDL, high density lipoproteins; TG, triglycerides; BUN, blood urea nitrogen; SCr, serum creatinine.

*P<0.05, **p<0.01, compared with SO rats.

^#^P<0.05, ^##^p<0.01, compared with UUO rats.
